#  A novel variant in the tropomyosin 3 gene presenting as an adult-onset distal myopathy - a case report

**DOI:** 10.1186/s12883-023-03225-3

**Published:** 2023-05-05

**Authors:** Zhiyong Chen, Monica Saini, Jasmine Shimin Koh, Gareth Zigui Lim, Nancy Jiaojiao Dang, Kalpana Prasad, Swee Hoon Koh, Karine Su Shan Tay, Ming Lee, Helen Lisa Ong, Yi Zhao, Ankit Tandon, Josiah Yui Huei Chai

**Affiliations:** 1 Department of Neurology, National Neuroscience Institute, 11 Jalan Tan Tock Seng, Singapore, 308433 Singapore; 2 Neuromuscular Laboratory, National Neuroscience Institute, Singapore, Singapore; 3 Department of Pathology, Singapore General Hospital, Singapore, Singapore; 4 Department of Clinical and Translational Research, Singapore General Hospital, Singapore, Singapore; 5 Department of Diagnostic Radiology, Tan Tock Seng Hospital, Singapore, Singapore

**Keywords:** Congenital myopathy, Distal myopathy, Tropomyosin, TPM3, Case report

## Abstract

**Background:**

We report a patient with a novel c.737 C > T variant (p.Ser246Leu) of the *TPM3* gene presenting with adult-onset distal myopathy.

**Case presentation:**

A 35-year-old Chinese male patient presented with a history of progressive finger weakness. Physical examination revealed differential finger extension weakness, together with predominant finger abduction, elbow flexion, ankle dorsiflexion and toe extension weakness. Muscle MRI showed disproportionate fatty infiltration of the glutei, sartorius and extensor digitorum longus muscles without significant wasting. Muscle biopsy and ultrastructural examination showed a non-specific myopathic pattern without nemaline or cap inclusions. Genetic sequencing revealed a novel heterozygous p.Ser246Leu variant (c.737C>T) of the *TPM3* gene which is predicted to be pathogenic. This variant is located in the area of the *TPM3* gene where the protein product interacts with actin at position Asp25 of actin. Mutations of *TPM3* in these loci have been shown to alter the sensitivity of thin filaments to the influx of calcium ions.

**Conclusion:**

This report further expands the phenotypic spectrum of myopathies associated with *TPM3* mutations, as mutations in *TPM3* had not previously been reported with adult-onset distal myopathy. We also discuss the interpretation of variants of unknown significance in patients with *TPM3* mutations and summarise the typical muscle MRI findings of patients with *TPM3* mutations.

**Supplementary Information:**

The online version contains supplementary material available at 10.1186/s12883-023-03225-3.

## Background

Inherited distal myopathies are a group of muscle disorders that selectively or predominantly involve distal skeletal muscles. It is a clinically, pathologically, and genetically heterogeneous group of diseases. At present, more than 25 genes have been reported to cause inherited distal myopathies [1].

The tropomyosin 3 (*TPM3*) gene encodes the alpha-tropomyosin, slow skeletal (α-tropomyosin_slow_, isoform Tpm3.12st), a protein expressed exclusively in type 1 (slow twitch) skeletal muscle fibres. Patients usually present with congenital to childhood-onset myopathies. Whilst infantile/childhood-onset distal muscle myopathy associated with *TPM3* mutations has been rarely reported [2], there are no prior reports of adult-onset distal muscle myopathy associated with *TPM3* mutations.

Here we report a patient with adult-onset distal myopathy with a novel c.737 C > T variant (p.Ser246Leu) of the *TPM3* gene and discuss its functional significance.

## Case presentation

The patient was a 35-year-old Chinese male, with an unremarkable antenatal, perinatal and developmental history. He was physically active, and completed military service uneventfully at 20 years of age. He first developed symptoms in his early 20s, when he experienced gradually progressive difficulty extending his fingers, leading to difficulty in using his handphone. In his early 30s, he developed progressive difficulty lifting heavy objects, as well as distal lower limb weakness. Speech, swallowing and respiratory functions were normal.

There was no parental consanguinity. His biological father had upper and lower limb weakness; patient is estranged from his biological father and could not provide further information. His biological mother and younger siblings (half-brother and half-sister, aged 33 and 29 respectively) were well. He was married, with no children.

**Fig. 1:**
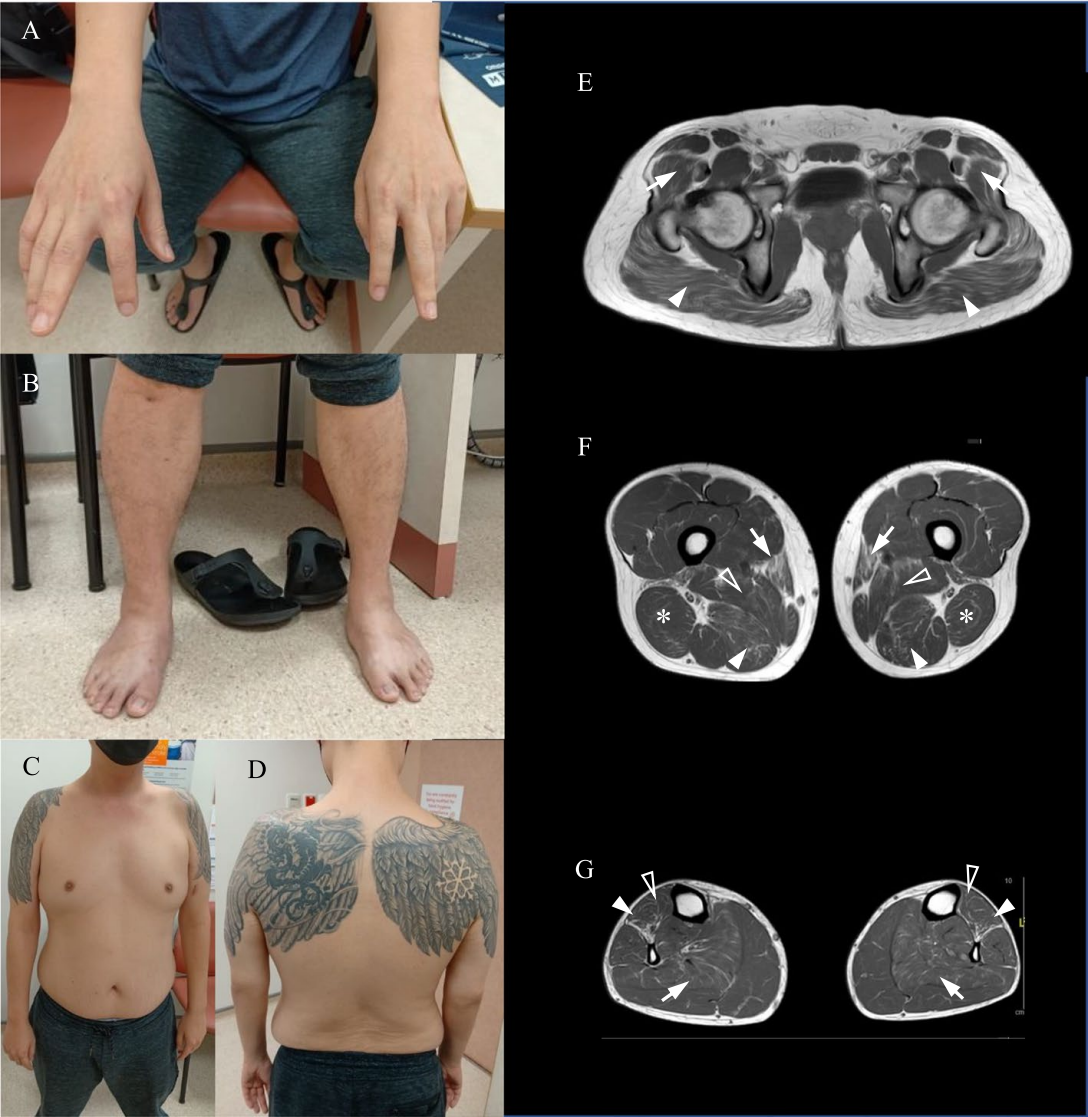
Physical examination findings and lower body MRI of patient. Differential asymmetric distal upper limb weakness of finger extensors and finger abductors (**A**). No significant atrophy is observed (**B**-**D**). Muscle MRI T1 sequence findings of patient at the level of the pelvic girdle (**E**), thighs (**F**) and calves (**G**). There is no significant loss of muscle volume or presence of muscle edema. **E** Mild symmetric fatty infiltration noted of the bilateral gluteus maximus (arrowhead) and gluteus medius (arrow). **F** Moderate fatty infiltration of the bilateral sartorius (solid arrowhead), mild infiltration of the adductor magnus (hollow arrowhead), posterior compartment of the thigh, particularly the bilateral semimembranosus (arrow) and biceps femoris (*). **G** Moderate fatty infiltration of the bilateral extensor digitorum longus muscles (solid arrowhead), mild fatty infiltration of bilateral tibialis anterior (hollow arrowhead), as well as mild fatty infiltration of the bilateral soleus muscles (arrow)

On examination, extraocular, facial, and bulbar muscle movements were normal. Muscle bulk was normal, with no wasting or hypertrophy observed. Muscle tone and deep tendon reflexes were also normal. Distal predominant muscle weakness was noted, most prominent in the bilateral index finger and left fourth and fifth finger extensors where differential weakness was seen (MRC grade 1–2; Fig. 1A-D). The distribution of weakness in the other muscle groups was as follows: In the upper limbs, finger abduction (MRC grade 2), elbow flexion (MRC grade 3), finger flexion and thumb abduction (MRC grade 4), wrist flexion and extension (MRC grade 5), shoulder abduction and elbow extension (MRC grade 4). In the lower limbs, ankle dorsiflexion (MRC grade 3), toe extension (MRC grade 3), knee flexion (MRC grade 5), Knee extension (MRC grade 4), hip flexion and extension (MRC grade 4). Mild muscle weakness was noted in axial muscles (neck flexion MRC grade 3, neck extension 4). Spine flexion and extension were intact. Sensory examination was normal. There were no skin, joint, or spinal abnormalities, and no tendon contractures seen.

Serum creatine kinase was normal. Nerve conduction study was unremarkable, while needle electromyography (EMG) revealed myopathic changes as characterized by low amplitude, short duration, polyphasic motor unit potentials with early recruitment, increased insertion activities without fibrillation potentials or positive sharp waves. Pertinent findings of the lower body muscle MRI (Magnetic resonance imaging; encompassing the pelvis, thighs and calves; Fig. 1E-G) included: fatty infiltration in gluteus maximus/ medius, sartorius, adductor magnus, semimembranosus, biceps femoris, extensor digitorum longus, tibialis anterior, and soleus. Other than the sartorius and extensor digitorum longus, whereby fatty infiltration was moderate, involvement of other affected muscles was mild. No significant loss of muscle volume or muscle oedema were evident. An electrocardiogram showed normal sinus rhythm, and transthoracic echocardiogram was normal.

**Fig. 2:**
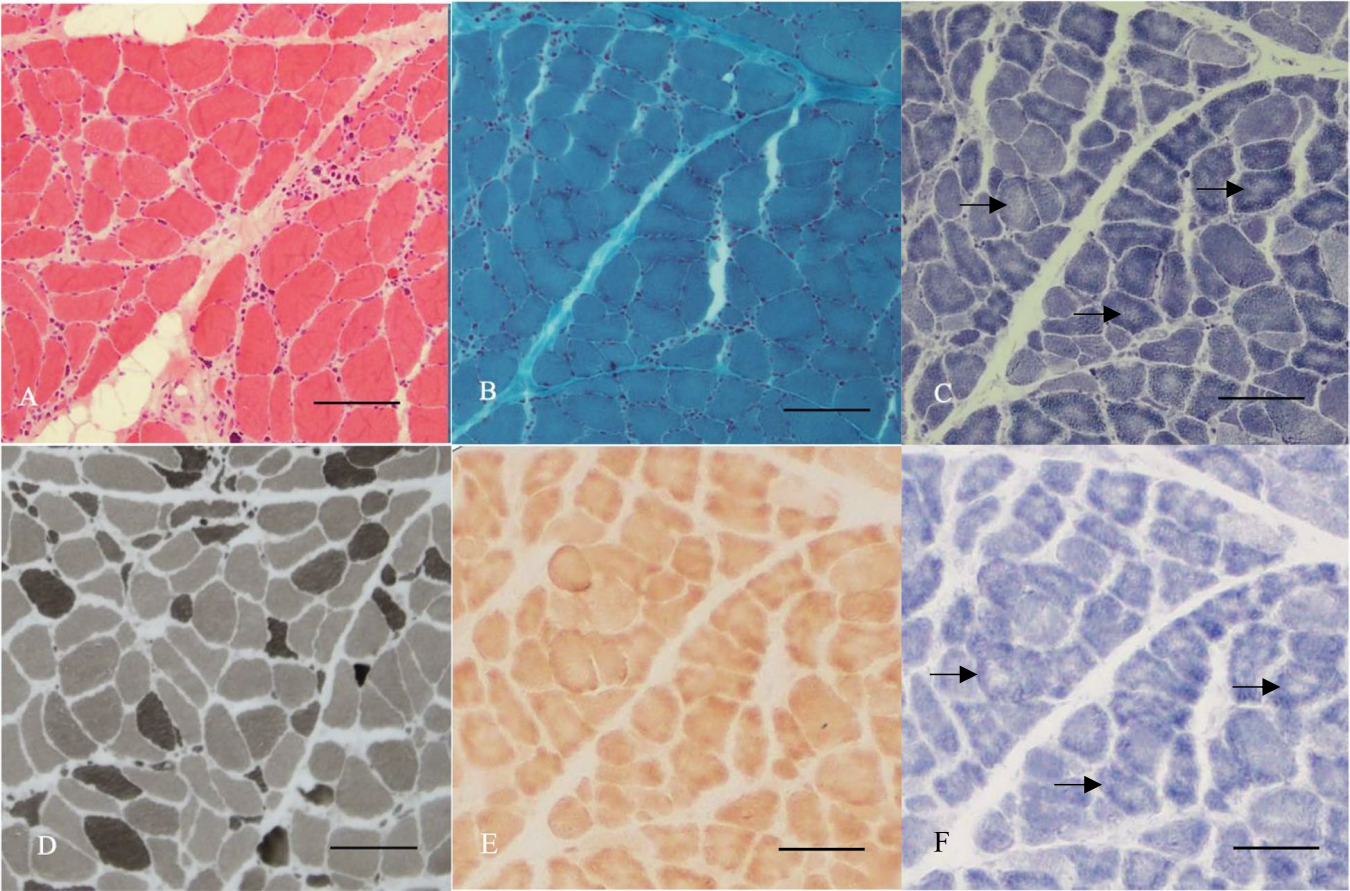
Biceps brachii muscle biopsy from patient. **A** There is variation in fibre size consistent with a chronic myopathy. **B** No inclusions are seen on Gömöri-trichrome stain. **C** and **F** Some fibres showed pale central areas on NADH stain and SDH stains (arrows) but not on **E** COX staining, suggesting regions with reduced oxidative enzyme activities. **D** There is predominantly type 2 fibre atrophy with no fibre type predominance. (**A**: Hematoxylin and eosin (HE) stain, original magnification × 10; **B**: Gömöri-trichrome stain, original magnification × 20; **C**: NADH-TR stain, original magnification × 10; **D**: ATPase pH9.4, original magnification × 10; **E**: COX stain, original magnification × 10; **F**: SDH stain, original magnification × 10; **A**-**F**: scale bar 100mm; picture resolution **A**-**F**: 1280 x1024)

**Fig. 3:**
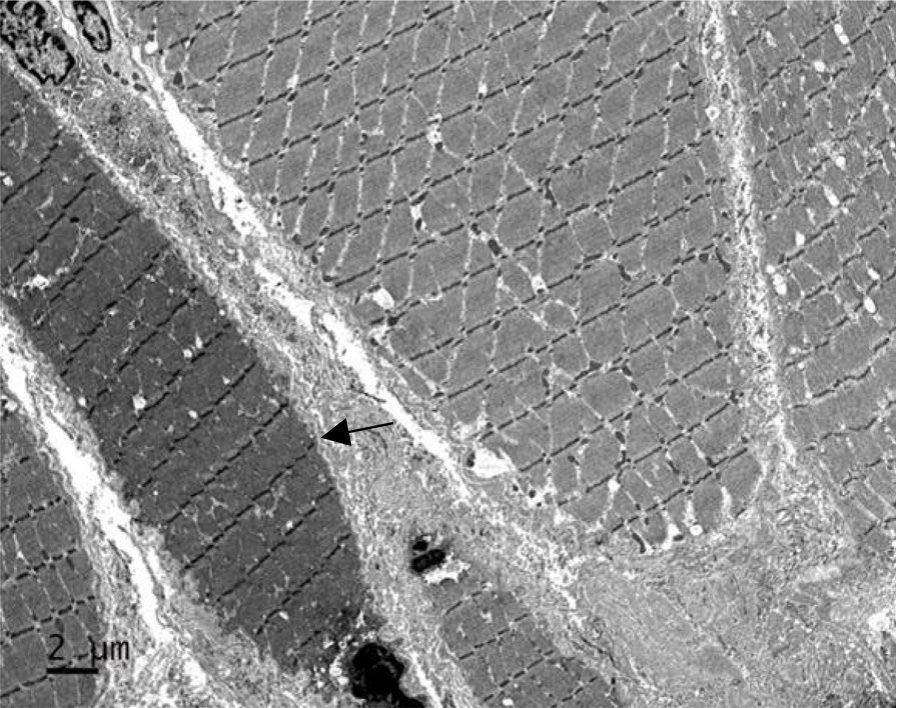
Electron microscopy revealed fibre atrophy (arrow) without the presence of nemaline rods (scale bar 2µm)

Biceps brachii muscle biopsy (Fig. 2; light microscope model: NI-U, Nikon, camera model: Nikon imaging device, DS-Camera, controller: DS-03, DS-Fil camera software: NIS-Elements BR4.2; Electron microscope model: JEOL JEM2100, camera model: Gatan SC1000 CCD camera, acquisition software: Gatan Microscopy Suite Digital micrograph) showed variation of muscle fibre size (Fig. 2A). Scattered and clustered predominantly atrophic type 2 fibres, together with nuclear clumps were seen (Mean type 1 fibre diameter: 30 ± 13 μm, mean type 2 fibre diameter 19 ± 13 μm, type 2 fibre percent 48%, type 2 factor atrophy factor 2.0) (Fig. 2D). Focal areas of endomysial and perimysial fibrosis, and increased adipose tissue were noted. Some fibres showed pale central areas on NADH (Fig. 2C) and SDH stains (Fig. 2F) but not on COX stain (Fig. 2E), suggesting regions with reduced oxidative enzyme activities. No necrosis or regenerating fibers were seen. There was no fibre type predominance. No abnormal inclusions were seen on Gömöri-trichrome stain (Fig. 2B). Electron microscopy showed atrophic fibers without the presence of nemaline rods (Fig. 3).

Genetic testing revealed a heterozygous p.Ser246Leu variant (NM_152263.4, c.737 C > T) in the *TPM3* gene. No other pathogenic variants in other genes known to be associated with genetic muscle diseases were identified. Cascade testing could not be performed, as patient’s biological father was not contactable. Reverse transcription-polymerase chain reaction (RT-PCR) analysis of the c.737 C > T variant was inconclusive, showing overexpression of the *TPM3* gene in muscle tissue as well as multiple regions of mRNA mis-splicing.

## Discussion and conclusions

We describe a patient with adult-onset distal myopathy with a novel c.737 C > T variant of the *TPM3* gene. Our report further expands the phenotypic spectrum associated with mutations in the *TPM3* gene and expands the genetic spectrum of disorders that result in adult-onset distal myopathy.

**Fig. 4:**
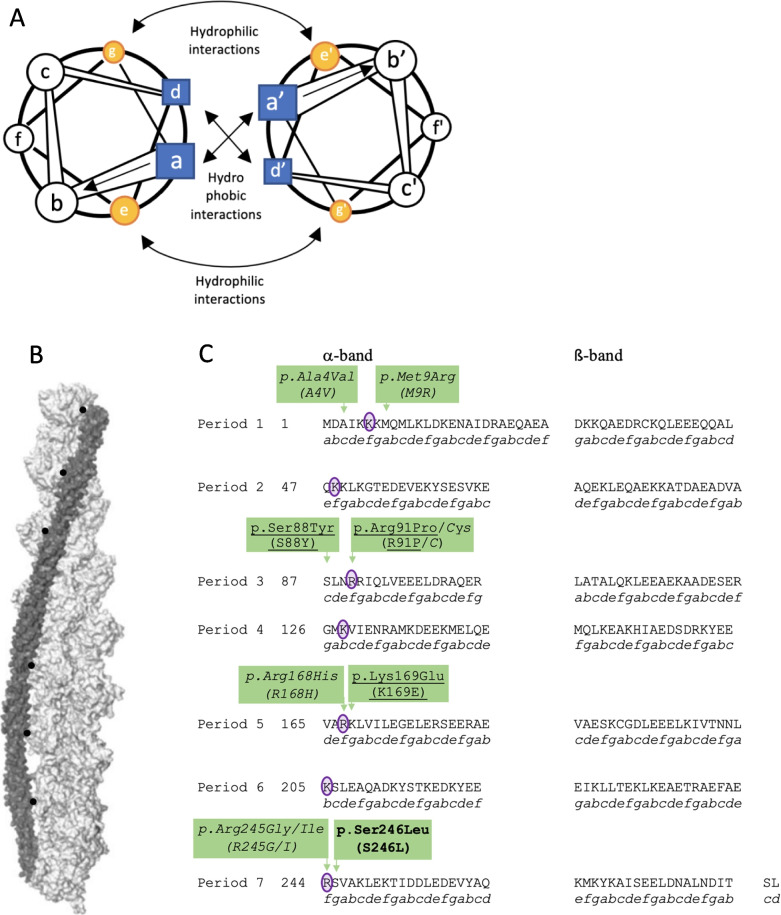
**A **Tropomyosins form α-helical coiled-coil dimers via a seven residue repeat motif in their amino acid sequence [*a-b-c-d-e-f-g*] [3]. This structure is stabilised by the hydrophobic interactions between *a-a’* and* d-d’* (shaded squares) together with the hydrophilic interactions between *g-e’* and *e-g’* (shaded circles) of the two tropomyosin chains. Actin binds to tropomyosin electrostatically at two points, at actin position Asp25 and a cluster of amino acids that includes actin at position Lys326. **B** The 3-dimensional structure of one of the two tropomyosin molecules (dark grey) interfacing with the actin double helix (light grey) at actin at position Asp25 (black) is shown here. **C** The α﻿-tropomyosin_slow_ amino acid sequence (annotated using the 1-letter amino acid abbreviation) is depicted here. The position of the amino acid in relation to the seven residue repeat motif of tropomyosin is indicated directly below the amino acid sequence. The structure of tropomyosin is further divided into seven quasi-repeating periods (periods 1-7) and α﻿-bands and ß-bands. The purple circles highlight residues interacting with actin at position Asp25. Known pathogenic variants of the *TPM3* gene that interact with actin at position Asp25 that result in an alteration of calcium ion sensitivity are shown here. Pathogenic variants that increase calcium ion sensitivity are written are underlined, while those that decrease Ca^2+^ sensitivity are italicised (annotations using their 1 letter amino acid abbreviation in brackets). The variant observed in our patient, p.Ser246Leu (S246L) is written in bold.

Tropomyosin polymerizes to form α-helical coiled-coil dimers. Its structure is conferred by a seven residue repeat motif [*a-b-c-d-e-f-g*] (Fig. 4A, C) [3]. Residues at positions *a* and *d* in the repeat are typically hydrophobic, creating a hydrophobic pocket between two tropomyosin chains. Polar residues at positions *g* and *e* stabilize the dimer through inter-helical salt bridges (Fig. 4A). Tropomyosin, together with troponin, associates along the entire length of actin filaments to form thin filaments. In skeletal muscle, tropomyosin interacts with troponin to regulate the calcium ion-mediated actin-myosin cross-bridge cycling that occurs during muscle contraction. At rest, tropomyosin interacts with actin in the “off” state conformation. The release of intra-sarcoplasmic calcium leads to a conformational change in the tropomyosin-troponin complex to an “on” state, uncovering the myosin-binding site of actin, allowing for the engagement of the myosin head with actin [4, 5]. The interaction of myosin heads with the actin filament leads to the sliding of the myosin filament along the actin filament, resulting in muscle contraction.

Tropomyosin is predicted to electrostatically interact with actin at position Asp25 of actin (Fig. 4B) as well as various other positions [6]. Variants of the *TPM3* gene that result in substitution of amino acid at the residues as well as the adjacent residues that bind with actin at position Asp25 have been shown in-vitro to alter the sensitivity of the tropomyosin-troponin complex to calcium ions and result phenotypically in myopathy [7]. These include the following variants: p.Ala4Val, p.Met9Arg, p.Arg91Cys, p.Arg168His, p.Lys169Glu, p.Arg245Gly and p.Arg245Ile (Fig. 4C) [7–9]. No benign variants have so far been reported in these Asp25 actin-binding sites.

Given the highly specific functional domains associated with the structure of the tropomyosin protein, there is a high rate of missense variants causing disease (44 of 46 non-VUS missense variants in the gene are pathogenic = 95.7%) as well as a low rate of benign missense variants.

In our patient, a p.Ser246Leu variant has been found in the *TPM3* gene. This variant has been reported in ClinVar (variant ID: 531,178) as a variant of unknown significance. This variant has not been reported in control databases, including GnomAD and ExAC. This variant results in the substitution of serine, an uncharged polar amino acid, with leucine, a hydrophobic aliphatic amino acid. The serine residue is a highly conserved amino acid position (PhasCons100way score: 1.00 and Phylo100way score: 8.117). The serine amino acid residue corresponds with position *g* of the seven amino acid repeat motif of tropomyosin. The p.Ser246Leu substitution results in the replacement of a polar residue with a hydrophobic residue. This is predicted to result in the disruption of the hydrophilic salt bridge between the tropomyosin monomers. Additionally, it lies adjacent to Arg245, an Asp25 actin-binding site. This amino acid change is thus also predicted to alter the sensitivity of the tropomyosin-troponin complex to calcium ions. As such, this variant is predicted in-silico to be damaging (CADD 24.5, EIGEN 0.448, FATHMM-MKL 0.9944, FATHMM-XF 0.9431, M-CAP 0.07326, MVP 0.9022, MutationTaster 1, Polyphen-2 0.847, PrimateAI 0.8751, PROVEAN: damaging, SIFT 0.033). Based on the above information, this variant is reclassified as pathogenic based on the ACMG criteria.

Muscle MRI studies of only six patients with *TPM3* mutations have been previously reported [10–13] (Table 1). Muscle MRI findings of our patient are similar to findings reported in prior studies, wherein changes are largely mild and involve fatty infiltration without significant muscle atrophy or edema. Fatty infiltration generally involves the gluteal muscles of the pelvic girdle, posterior compartment of the thigh, anterior compartment and the soleus muscle (with sparing of the gastrocnemius) of the calf. Other reported muscle involvement, beyond the lower limbs, include the involvement of the paraspinal muscles, which were not evaluated in our patient.

**Table 1:** Summary of muscle MRI findings based on published literature. Muscles affected by fatty infiltration are indicated in boxes. Severity of involvement is indicated in brackets. Boxes are kept blank if no involvement is reported

	Patient num-ber	Pelvic girdle	Thigh			Calf			Others
			Anterior	Medial	Posterior	Anterior	Lateral	Posterior	
Kiphuth [10]	1	gluteus	generalised	generalised	generalised	generalised	generalised	generalised	erector spinae, oblique, rectus abdominis
	2	insufficient information							
Moreno [13]	3		sartorius	gracilis	more in biceps femoris(mild)	(mild)		soleus (mild)	paraspinal
Munot [12]	4		(mild)	(mild)	(mild)				
Schreckenbach [11]	5	gluteus maximus + gluteus minimus			semimembranosus, biceps femoris	tibialis anterior		soleus	paraspinal, hypertrophy of masseter
	6	gluteus minimus						soleus	hypertrophy of masseter
Our patient		gluteus maximus (mild) gluteus minimus (mild)	sartorius (moderate)	adductor magnus (mild)	semimembranosus (mild) biceps femoris (mild)	extensor digitorum longus (moderate), tibialis anterior (mild)		soleus (mild)	

Interestingly, we observed disproportionate involvement of the extensor digitorum longus, which correlated with disproportionate toe extension weakness, indicating focal involvement within one muscle compartment. This finding was previously reported in only one patient with *TPM3* mutation [11]. While focal involvement was also reflected clinically by the presence of disproportionate muscle weakness of the first dorsal interossei and extensor digitorum longus, this could not be verified radiologically as MRI of the upper limbs was not performed. Further descriptive studies with larger patient cohorts are required for elucidation of typical patterns of muscle involvement in patients with *TPM3* mutations.

Type 1 fibre hypotrophy, commonly reported in patients with *TPM3* mutations, is postulated to occur because of the exclusive expression of α-tropomyosin_slow_ in type 1 muscle fibres [14]. Type 1 fibre hypotrophy is however neither ubiquitous nor unique in patients with *TPM3* mutations [10, 11, 15–17], it is also common in patients with *TPM2* mutations, despite the expression of tropomyosin beta chain in both fibre types. Our patient did not demonstrate Type 1 fibre hypotrophy. While type 2 fibre atrophy may be secondary to other medical co-morbidities or disuse atrophy, no such conditions were noted in our patient. Cap structures and nemaline bodies appear to be more frequently observed in histological specimens taken at older ages. This has been postulated to be due to a protracted disease process leading to the accumulation of sarcomeric protein inclusions over time [8]. The absence of cap structures and nemaline bodies is possibly related to the adult onset of illness and the short duration of disease. Better understanding of the pathophysiological underpinnings of how mutations in the *TPM3* gene leads to the above histological changes is required.

There are some limitations to our report. Firstly, muscle MRI of the upper extremity and trunk was not performed for this patient; thus, we were unable to characterize the full extent of muscle involvement in this patient. Additionally, functional studies quantifying contractility dysfunction associated with this variant have not been performed.

In conclusion, our report further expands the phenotypic spectrum associated with *TPM3* mutations and adds to the repertoire of genetic disorders reported to cause adult-onset inherited distal myopathy. We also discuss the interpretation of variants of unknown significance in patients with *TPM3* mutations and reviewed the typical muscle MRI findings of patients with *TPM3* mutations.

## Supplementary Information

**Additional file 1.**

## Data Availability

The datasets generated and/or analysed during the current study are available in the ENA repository, Accession number: PRJEB57294.

## Abbreviations

a-tropomyosin_slow_: alpha-tropomyosin, slow skeletal
ACMG: American college of medical geneticists
COX: Cytochrome c oxidase
DNA: Deoxyribonucleic acid
EMG: Electromyography
MRC: Medical research council
MRI: magnetic resonance imaging
NADH-TR: Nicotinamide adenine dinucleotide- tetrazolium reductase
SDH: Succinate dehydrogenase
TPM3: Tropomyosin 3

## References

[1] Milone M, Liewluck T (2019). The unfolding spectrum of inherited distal myopathies. Muscle Nerve.

[2] Ilkovski B, Mokbel N, Lewis RA, Walker K, Nowak KJ, Domazetovska A (2008). Disease severity and thin filament regulation in M9R TPM3 nemaline myopathy. J Neuropathol Exp Neurol.

[3] McLachlan AD, Stewart M (1975). Tropomyosin coiled-coil interactions: evidence for an unstaggered structure. J Mol Biol.

[4] Gordon AM, Homsher E, Regnier M (2000). Regulation of contraction in striated muscle. Physiol Rev.

[5] Tobacman LS (1996). Thin filament-mediated regulation of cardiac contraction. Annu Rev Physiol.

[6] Li XE, Tobacman LS, Mun JY, Craig R, Fischer S, Lehman W (2011). Tropomyosin position on F-actin revealed by EM reconstruction and computational chemistry. Biophys J.

[7] Marston S, Memo M, Messer A, Papadaki M, Nowak K, McNamara E (2013). Mutations in repeating structural motifs of tropomyosin cause gain of function in skeletal muscle myopathy patients. Hum Mol Genet.

[8] Marttila M, Lehtokari V-L, Marston S, Nyman TA, Barnerias C, Beggs AH (2014). Mutation update and genotype-phenotype correlations of novel and previously described mutations in TPM2 and TPM3 causing congenital myopathies. Hum Mutat.

[9] Yuen M, Cooper ST, Marston SB, Nowak KJ, McNamara E, Mokbel N (2015). Muscle weakness in TPM3-myopathy is due to reduced Ca2+-sensitivity and impaired acto-myosin cross-bridge cycling in slow fibres. Hum Mol Genet.

[10] Kiphuth IC, Krause S, Huttner HB, Dekomien G, Struffert T, Schröder R (2010). Autosomal dominant nemaline myopathy caused by a novel alpha-tropomyosin 3 mutation. J Neurol.

[11] Schreckenbach T, Schröder JM, Voit T, Abicht A, Neuen-Jacob E, Roos A (2014). Novel TPM3 mutation in a family with cap myopathy and review of the literature. Neuromuscul Disord.

[12] Munot P, Lashley D, Jungbluth H, Feng L, Pitt M, Robb SA (2010). Congenital fibre type disproportion associated with mutations in the tropomyosin 3 (TPM3) gene mimicking congenital myasthenia. Neuromuscul Disord.

[13] Moreno CAM, Estephan E, de Fappi P, Monges A, Lubieniecki S, Lopes Abath Neto F (2020). Congenital fiber type disproportion caused by TPM3 mutation: a report of two atypical cases. Neuromuscul Disord.

[14] Perry SV (2001). Vertebrate tropomyosin: distribution, properties and function. J Muscle Res Cell Motil.

[15] Donkervoort S, Papadaki M, de Winter JM, Neu MB, Kirschner J, Bolduc V (2015). TPM3 deletions cause a hypercontractile congenital muscle stiffness phenotype. Ann Neurol.

[16] Ohlsson M, Fidzianska A, Tajsharghi H, Oldfors A (2009). TPM3 mutation in one of the original cases of cap disease. Neurology.

[17] Pénisson-Besnier I, Monnier N, Toutain A, Dubas F, Laing N (2007). A second pedigree with autosomal dominant nemaline myopathy caused by TPM3 mutation: a clinical and pathological study. Neuromuscul Disord.

